# Higher habitual protein intake is not linked to decline of iohexol-measured GFR in adults ≥40 years

**DOI:** 10.1093/ckj/sfag235

**Published:** 2026-07-11

**Authors:** Ludvig Balteskard Rinde, Laila A Hopstock, Marie W Lundblad, Nikoline Balteskard Rinde, Karl-Marius Brobak, Jon Viljar Norvik, Inger-Therese Enoksen, Marit D Solbu, Ole-Martin Fuskevåg, Juan-Jesus Carrero, Monica Hauger Carlsen, Bjørn Odvar Eriksen, Toralf Melsom

**Affiliations:** Metabolic and Renal Research Group, Department of Clinical Medicine, UiT The Arctic University of Norway, Tromsø, Norway; Section of Nephrology, Department of Medicine, University Hospital of North Norway, Tromsø, Norway; Department of Health and Care Sciences, UiT The Arctic University of Norway, Tromsø, Norway; Department of Community Medicine, UiT The Arctic University of Norway, Tromsø, Norway; Metabolic and Renal Research Group, Department of Clinical Medicine, UiT The Arctic University of Norway, Tromsø, Norway; Section of Nephrology, Department of Medicine, University Hospital of North Norway, Tromsø, Norway; Metabolic and Renal Research Group, Department of Clinical Medicine, UiT The Arctic University of Norway, Tromsø, Norway; Section of Nephrology, Department of Medicine, University Hospital of North Norway, Tromsø, Norway; Metabolic and Renal Research Group, Department of Clinical Medicine, UiT The Arctic University of Norway, Tromsø, Norway; Section of Nephrology, Department of Medicine, University Hospital of North Norway, Tromsø, Norway; Metabolic and Renal Research Group, Department of Clinical Medicine, UiT The Arctic University of Norway, Tromsø, Norway; Section of Emergency Medicine, Department of Medicine, University Hospital of North Norway, Tromsø, Norway; Metabolic and Renal Research Group, Department of Clinical Medicine, UiT The Arctic University of Norway, Tromsø, Norway; Section of Nephrology, Department of Medicine, University Hospital of North Norway, Tromsø, Norway; Department of Laboratory Medicine, University Hospital of North Norway, Tromsø, Norway; Tromsø Endocrine Research Group, Department of Clinical Medicine, UiT The Arctic University of Norway, Tromsø, Norway; Department of Medical Epidemiology and Biostatistics, Karolinska Institutet, Stockholm, Sweden; Division of Nephrology, Department of Clinical Sciences, Karolinska Institutet, Danderyd Hospital, Stockholm, Sweden; Department of Nutrition, Institute of Basic Medical Sciences, Faculty of Medicine, University of Oslo, Oslo, Norway; Metabolic and Renal Research Group, Department of Clinical Medicine, UiT The Arctic University of Norway, Tromsø, Norway; Section of Nephrology, Department of Medicine, University Hospital of North Norway, Tromsø, Norway; Metabolic and Renal Research Group, Department of Clinical Medicine, UiT The Arctic University of Norway, Tromsø, Norway; Section of Nephrology, Department of Medicine, University Hospital of North Norway, Tromsø, Norway

**Keywords:** chronic kidney disease, epidemiology, GFR decline, nephrology, nutrition

## Abstract

**Background:**

High habitual dietary protein intake may increase glomerular workload and contribute to hyperfiltration, but the long-term renal effects in individuals without chronic kidney disease remain debated. Prior population studies have relied on estimated glomerular filtration rate, which may be biased by non-GFR determinants. We examined whether higher protein intake is associated with decline in kidney function using repeated measurements of iohexol-measured GFR (mGFR).

**Methods:**

We analysed data from the Renal Iohexol Clearance Survey, a population-based cohort with repeated mGFR measurements over follow-up (*n* = 1324). Baseline protein intake was estimated using a validated food-frequency questionnaire. Associations of protein intake with GFR decline rate, accelerated GFR decline (top 10% with the steepest annual reductions), and incident mGFR <60 ml/min/1.73 m² were assessed using linear mixed models, logistic regression, and interval-censored Cox regression.

**Results:**

Mean age was 64 years, 50% were women, and mean baseline mGFR was 89 ml/min/1.73 m². Median follow-up was 10 years. Mean reported protein intake was 1.2 ± 0.5 g/kg/day, with the lowest quartile consuming <0.9 g/kg/day and the highest ≥1.4 g/kg/day. Protein intake was not associated with mGFR decline [−0.01 ml/min/1.73 m² per year (95% CI −0.04 to 0.02) per 0.1 g/kg/day higher protein intake]. Protein intake was not associated with accelerated GFR decline or incident mGFR <60 ml/min/1.73 m².

**Conclusions:**

Among middle-aged and older adults in the general population, higher estimated habitual protein intake was not associated with mGFR decline.

## Graphical Abstract

**Graphical Abstract:**
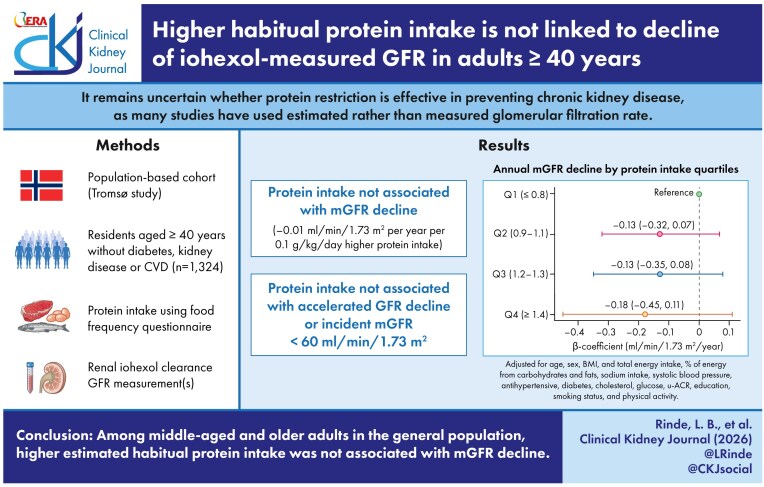

KEY LEARNING POINTS **What was known:**

- High-protein diets may increase intraglomerular pressure and hyperfiltration.
- While reducing protein intake is a guideline-recommended practice in advanced CKD to potentially slow disease progression by preventing glomerular overload, it remains unclear whether protein restriction is a useful strategy for preventing CKD.
- Previous population studies have mainly relied on estimated rather than measured glomerular filtration rate.

**This study adds:**

- Higher habitual protein intake was not associated with faster decline in iohexol-measured glomerular filtration rate in this general population cohort.
- Higher protein intake was not associated with accelerated kidney function decline or incident mGFR <60 ml/min/1.73 m².
- The findings were consistent across quartile, interaction, and sensitivity analyses.

**Potential impact:**

- These findings do not support habitual protein intake within commonly consumed ranges as a major target for primary prevention of chronic kidney disease.
- The results may help clinicians counsel adults without chronic kidney disease about moderate-to-high habitual protein intake.

## INTRODUCTION

Age-related decline in glomerular filtration rate (GFR) is a major driver of chronic kidney disease (CKD) burden, leading to a high prevalence of CKD among older adults [1]. Established risk factors such as hypertension, obesity, and diabetes are well-known contributors to CKD, yet much of the variation in GFR decline remains unexplained [2]. This highlights the need to identify modifiable risk factors and evidence-based lifestyle strategies to prevent CKD. While dietary interventions are widely recommended for cardiovascular disease (CVD) prevention [3], dietary recommendations for CKD prevention remain sparse and are largely extrapolated from CVD-focused studies [4].

Nephron number declines with age even in otherwise healthy kidneys, without necessarily increasing single-nephron GFR. However, lower nephron number may reduce renal reserve and increase susceptibility to additional hyperfiltration stimuli, such as obesity [5, 6]. High-protein diets have been shown to increase intraglomerular pressure and induce hyperfiltration [7, 8]. The renal effects of protein intake may also differ by protein source, as meat, dairy, and plant-derived proteins may differ in acid load and renal haemodynamic effects [9, 10]. While reducing protein intake is a guideline-recommended practice in advanced CKD to potentially slow disease progression by preventing glomerular overload [11], it remains unclear whether protein restriction is a useful strategy for preventing CKD [4].

Protein intake enhances muscle protein synthesis, and many athletes use protein supplementation to optimize endurance and resistance training [12]. Furthermore, high-protein diets are increasingly popular for weight loss [13], and adequate protein intake is often emphasized for older adults to help reduce decline in muscle mass [14]. Recently, the Dietary Guidelines for Americans highlighted the importance of protein intake [15]. These competing considerations underscore the importance of clarifying the potential role of protein intake as a modifiable risk factor for CKD [16]. While some population-based studies suggest that high protein intake may accelerate GFR decline and increase CKD risk [17–20], others have found a protective effect [8, 9, 21–23] or no association in individuals without CKD [10, 24]. A major limitation of previous research is the reliance on creatinine-based GFR estimates (eGFR), which may be influenced by non-GFR determinants. Higher muscle mass may increase endogenous creatinine generation, whereas dietary protein may also influence serum creatinine independently of true GFR [25, 26].

To address this gap in evidence, we investigated the association between dietary protein intake and GFR decline in the Renal Iohexol Clearance Survey (RENIS), a unique general population cohort with repeated iohexol-based GFR measurements (mGFR) over long-term follow-up, to determine whether higher habitual protein intake is associated with age-related GFR decline.

## MATERIALS AND METHODS

### Study population

The Tromsø Study is an ongoing population-based cohort with Tromsø7 (2015–16) being the latest [27]. In Tromsø7, all residents aged ≥40 years were invited, with 65% (*n* = 21 083) participating. All participants were asked to complete a food frequency questionnaire (FFQ), and 72% (*n* = 15 146) returned it.

RENIS is a sub-study of the Tromsø Study. Detailed information on the design, sample, and data collection of the RENIS cohort is available in previous publications [28–30]. The baseline study, RENIS-T6 (2007–09), included 1627 women and men aged 50–62 years, invited from Tromsø6 without self-reported diabetes, kidney disease, or CVD. Follow-ups were conducted in RENIS-FU (2013–15), RENIS-3 (2018–20), and RENIS-4 (2024–25). In RENIS-4, all participants who had previously attended RENIS and were still alive were invited to participate.

**Figure 1:**
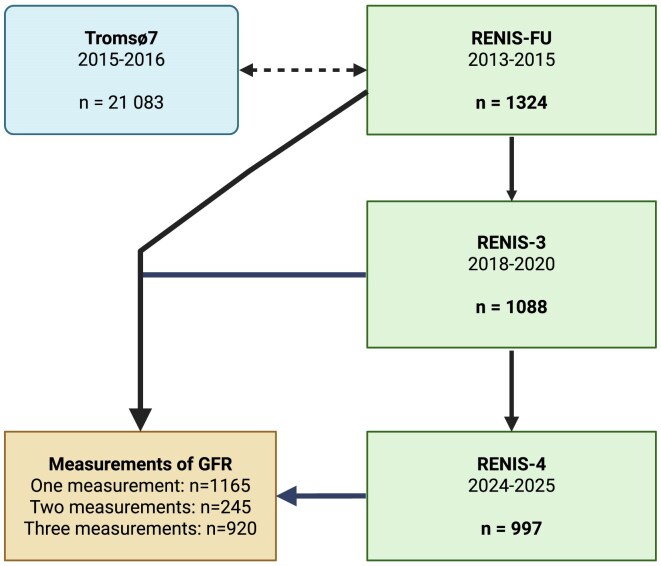
Study participants in the Renal Iohexol Clearance Survey (RENIS). Participants with three mGFR measurements attended RENIS-FU, RENIS-3, and RENIS-4. Participants with two mGFR measurements attended RENIS-FU and one follow-up visit. RENIS-FU, RENIS-Follow-up.

Data collection in Tromsø7, which included FFQ data, overlapped with RENIS-FU, and RENIS-FU served as the study baseline. Tromsø7 was conducted shortly after RENIS-FU. A total of 1324 participants attended RENIS-FU, with 1088 attending RENIS-3 and 997 attending RENIS-4 (Fig. 1). A total of 1165 participants had at least one follow-up GFR measurement; 920 had three total mGFR measurements and 245 had two total mGFR measurements.

Although those with diabetes, kidney disease, or CVD were excluded from RENIS-T6, some developed these conditions by the time of RENIS-FU. Diabetes was classified using self-report, medication use, and/or laboratory criteria: HbA1c ≥6.5%, fasting glucose ≥7 mmol/l. CVD included myocardial infarction, stroke, coronary revascularization, artery stenosis diagnosis, or sudden death without a non-CVD cause.

The study was approved by the Regional Committee for Medical and Health Research Ethics in North Norway (REK NORD 741313), and all participants gave informed written consent.

### Food frequency questionnaire

Protein intake was estimated from FFQ data in Tromsø7 [31]. The median interval between baseline iohexol measurement and FFQ completion was 20.5 months [interquartile range (IQR) 16.0–25.1]. The FFQ was developed at the University of Oslo to capture comprehensive dietary information, including the frequency and quantity of habitual intake over the past year for 261 dietary items [32]. It has been validated in Norwegian adults, supporting its use to estimate average energy and nutrient intakes [32–34]. The average daily protein intake was calculated and reported in grams (g) and grams per kilogram (kg) of body weight (g/kg), while daily energy intake was expressed in kilojoules (kJ). These calculations were performed using the KBS (Kostberegningssystem) food and nutrient calculation system (version 7.3), which utilizes the KBS food database (version AE14). The nutrient variables available for the present analysis included total protein and total energy intake, as well as macronutrient composition, derived from the KBS nutrient calculation system. Validated variables for animal- and plant-derived protein were not available in the analytic dataset.

### Iohexol clearance

GFR was measured using single-sample plasma iohexol clearance, as described in detail in the Supplementary Methods and previously in RENIS [29]. Briefly, participants were examined after fasting and were instructed to avoid large meals with meat and non-steroidal anti-inflammatory drugs for 2 days before the examination. They were reminded not to restrict water intake. The examination was rescheduled in case of acute illness or recent radiological contrast exposure.

### Baseline data and measurements

Baseline and follow-up data were collected at the Clinical Research Unit of the University Hospital of North Norway, as previously described [28]. Serum creatinine was measured using an enzymatic assay standardized to isotope-dilution mass spectrometry at the same study visit as the iohexol clearance measurement. One serum creatinine measurement was used at each RENIS examination [30]. The inter-assay coefficient of variation over the entire study period was 2.3% for serum creatinine levels and <3.2% for cystatin C. Three first-morning urine samples were collected on different days before GFR measurement, and the median urinary albumin-to-creatinine ratio (u-ACR) was used.

Hypertension was defined as a mean systolic blood pressure ≥140 mmHg, a diastolic pressure ≥90 mmHg, or self-reported use of antihypertensives. Hyperfiltration was defined according to previous RENIS analyses as absolute GFR above the 90th percentile after adjustment for sex, age, weight, height, and use of renin–angiotensin system inhibitors, corresponding to GFR >127.1 ml/min. Prediabetes was defined as fasting plasma glucose of 5.6–6.9 mmol/l according to the American Diabetes Association definition [35], and obesity was defined as BMI ≥30 kg/m². Smoking status was determined from a questionnaire and categorized as current vs previous/never smoker. Physical activity was assessed by engagement in at least one hour of moderate or vigorous activity weekly (yes/no). Educational attainment was categorized as ≥13 years (college or university) vs <13 years (high school or less).

### Statistical analysis

#### Baseline data

Baseline data are presented by quartiles of protein intake, with continuous variables expressed as means ± standard deviations (SD) for normally distributed data or as medians with IQR for skewed data. Categorical data are presented as counts and percentages. Comparisons between groups were assessed with linear regression for normally distributed data, the Kruskal-Wallis test for skewed data, and the chi-squared test for categorical variables.

#### Multiple imputation

In total, 233 participants (17.6%) had missing data on protein and energy intake, and 156 (11.8%) on education level (Supplementary Table S1). Missingness in these variables was assumed to be missing at random [31] and explained by observed characteristics included in the imputation model. Missing variables were imputed via multiple imputation with 50 imputed datasets [36]. Missing mGFR values during follow-up were not imputed, in accordance with the practice for linear mixed models [37].

#### Linear mixed models

The longitudinal relationship between mGFR and protein intake was assessed using linear mixed models with random intercepts and slopes and an unstructured covariance matrix. The estimation of individual mGFR slopes is described in detail in the supplementary materials. The effect of the independent variable on the change rate of GFR was analysed by including a two-way interaction between the independent variable and time [38]. A negative GFR change rate indicates a yearly mean reduction in GFR. Each participant’s annual GFR change rate (in ml/min per 1.73 m² per year) was calculated, with protein intake (in g/kg/day) as an independent variable, and 95% confidence intervals (CIs) were reported in separate models adjusted for known risk factors for CKD. Model 1 was unadjusted; Model 2 was adjusted for baseline age, sex, BMI, and total energy intake; and Model 3 included adjustments for the proportion of energy from carbohydrates and fats, total sodium intake, systolic blood pressure, use of antihypertensives (yes/no), diabetes mellitus (yes/no), total cholesterol, glucose levels, u-ACR, smoking status (yes/no), education (yes/no), and physical activity (yes/no).

We assessed non-linear relationships between protein intake and mGFR decline using mixed-effects models with quartiles and trend terms for repeated measurements. Sensitivity analyses employed GAMMs with smooth functions and random effects for intercept and slope to detect non-linear patterns.

#### Sensitivity analyses

Indexing GFR by body surface area has been criticized for failing to account for variations in body size and sex. This may obscure associations with hyperfiltration and introduce confounding by accounting for changes in body size over time in longitudinal studies [39]. To address these, we performed sensitivity analyses with absolute GFR (aGFR) in ml/min, adjusting for height and weight in Models 2 and 3. To account for baseline GFR and hyperfiltration from other conditions, we conducted analyses adjusting for baseline mGFR, and analyses including only participants with baseline diabetes, obesity, or CVD. To address potential inaccuracies in protein intake, we conducted sensitivity analyses by excluding the lowest and highest 1% of protein intake. Given the median interval of 20.5 months between the iohexol measurements and the completion of the FFQ, we conducted sensitivity analyses that included only participants with less than 12 months between these two assessments. Finally, we performed analyses using complete cases.

#### Multiple logistic regression

We used multivariable logistic regression to estimate the odds ratios (ORs) for the association between protein intake and accelerated GFR decline. Accelerated decline was defined as an annual GFR change rate below the 10th percentile (i.e. steeper than −2.08 ml/min/1.73 m² per year). The threshold corresponds to the 10th percentile of the estimated individual annual mGFR slopes within the study cohort, derived from a linear mixed model adjusted for CKD risk factors, as described [40, 41]. For participants with only one mGFR measurement, individual change rates were predicted using baseline covariates. In sensitivity analyses, we performed logistic regression on participants with at least two GFR measurements (*n* = 1165).

#### Interval-censored Cox regression

The exact timing of crossing the mGFR threshold of <60 ml/min/1.73 m² was interval-censored, occurring between two observations. Interval-censored Cox regression was used to explore associations between protein intake and incident mGFR <60 ml/min/1.73 m², with the event interval defined as the time from the last observation with mGFR ≥60 ml/min/1.73 m² to the first observation with mGFR <60 ml/min/1.73 m². Since multiple imputation does not support interval-censored models, we used the complete-case dataset (*n* = 1064). A model-based cumulative incidence plot for incident mGFR <60 ml/min/1.73 m² by protein-intake quartile was generated from the interval-censored Cox model (Supplementary Fig. S2).

The same adjustment models from the mixed-model analyses were used in multiple logistic and interval-censored Cox regressions. Sensitivity analyses adjusting for baseline mGFR were conducted.

#### Interaction analyses

We examined effect modification by sex, obesity, hypertension, and prediabetes, as these subgroups are more prone to hyperfiltration and may be more susceptible to the effects of higher protein intake [38]. Effect modification was assessed by including three-way interaction terms between time, protein intake, and each covariate in separate linear mixed-effects models.

We evaluated whether albuminuria modified the association between protein intake and mGFR decline by including a three-way interaction between time, protein intake, and u-ACR, categorized as below or above detection limit (<10 mg/g) [42]. Interactions between sex and protein intake for accelerated GFR decline and incident mGFR <60 ml/min/1.73 m² were examined using two-way interaction terms in logistic and interval-censored Cox regression models.

A *P*-value below .05 was considered statistically significant. All statistical analyses and the multiple imputation were conducted using STATA/MP software, version 19.0 (StataCorp LP, College Station, TX).

## RESULTS

### Baseline characteristics

The characteristics of all 1324 participants in RENIS-FU are shown in Table 1. The baseline mean age was 63.6 (SD 4.0) years, and 50.4% (*n* = 667) were women. The mean baseline mGFR was 89.1 ml/min/1.73 m² (SD 14.5). The median time between the first and last mGFR measurements was 10.0 years (IQR 8.9–10.6). At baseline, 27 (2.0%) participants had diabetes, 54 (4.1%) had previous CVD, and 33 (2.5%) had mGFR below 60 ml/min/1.73 m^2^.

**Table 1:** Baseline characteristics by quartiles of total protein intake (RENIS).

		Quartile of total protein intake, g/kg/d				
Variables	Total (*N* = 1324)	Q1 (<0.9)	Q2 (0.9–1.1)	Q3 (1.2–1.3)	Q4 (≥1.4)	*P*-values
Age, Y Mean (SD)	63.6 (4.0)	64.2 (3.9)	63.6 (4.0)	63.5 (4.0)	63.2 (3.9)	<.01
Male, *n* (%)	657 (49.6)	161 (48.5)	173 (52.5)	166 (50.2)	157 (47.5)	.61
BMI, Mean (SD)	27.1 (4.2)	28.7 (4.4)	27.5 (3.8)	26.7 (4.0)	25.7 (3.8)	<.01
Protein, g/kg/day, Mean (SD)	1.2 (0.5)	0.8 (0.1)	1.0 (0.1)	1.3 (0.1)	1.8 (0.5)	<.01
Energy, kJ/day, Mean (SD)	9321 (3584)	6412 (1643)	8323 (1628)	9555 (1908)	12 878 (4632)	<.01
Protein, E%, Mean (SD)	17.8 (2.7)	17.1 (3.1)	17.6 (2.4)	17.9 (2.5)	18.4 (2.8)	<.01
Fat, E%, Mean (SD)	33.6 (5.8)	32.4 (6.3)	33.3 (5.8)	33.8 (5.4)	34.9 (5.5)	<.01
Carbohydrates, E%, Mean (SD)	42.1 (6.4)	42.3 (7.2)	42.2 (6.2)	42.4 (6.1)	41.1 (5.8)	.02
mGFR, ml/min/1.73 m^2^, Mean (SD)	89.1 (14.5)	86.8 (14.7)	88.4 (14.1)	89.3 (14.1)	91.7 (14.5)	<.01
Creatinine, µmol/l, Mean (SD)	70.5 (13.7)	71.3 (13.7)	71.7 (14.0)	70.4 (13.5)	69.1 (13.6)	.03
Urine ACR, mg/g, Median (IQR)	0.3 (0.1–0.6)	0.3 (0.1–0.5)	0.3 (0.1–0.6)	0.3 (0.1–0.6)	0.3 (0.1–0.6)	.88
Systolic blood pressure, Mean (SD)	130.7 (17.0)	132.5 (16.4)	131.5 (16.4)	129.0 (17.6)	129.5 (17.1)	.02
Diastolic blood pressure, Mean (SD)	81.9 (9.3)	82.9 (9.1)	82.2 (9.0)	81.5 (9.8)	81.0 (9.2)	<.01
Total cholesterol, Mean (SD)	5.5 (1.0)	5.5 (1.0)	5.5 (0.9)	5.5 (1.0)	5.5 (1.0)	.77
Fasting glucose, Mean (SD)	5.5 (0.6)	5.6 (0.6)	5.5 (0.6)	5.5 (0.6)	5.4 (0.6)	<.01
Hypertension, *n* (%)^a^	690 (52.1)	192 (57.9)	186 (56.3)	161 (48.5)	151 (45.3)	<.01
mGFR <60 ml/min/1.73 m^2^, *n* (%)	33 (2.5)	13 (4.1)	12 (3.6)	6 (1.8)	2 (0.8)	.02
Hyperfiltration, *n* (%)^b^	133 (10.0)	36 (11.1)	30 (9.0)	33 (9.9)	34 (10.5)	.90
Cardiovascular disease, *n* (%)^c^	54 (4.1)	17 (5.2)	12 (3.6)	16 (4.8)	9 (2.6)	.33
Diabetes, *n* (%)	27 (2.0)	10 (3.0)	8 (2.5)	4 (1.1)	5 (1.5)	.11
Prediabetes, *n* (%)^d^	633 (47.8)	177 (53.5)	164 (49.5)	152 (46.0)	140 (42.3)	<.01
Obesity, *n* (%)^e^	287 (21.7)	108 (32.5)	77 (23.3)	64 (19.1)	38 (11.6)	<.01
Active smokers, *n* (%)	138 (10.4)	33 (10.0)	28 (8.3)	37 (11.2)	40 (12.0)	.04
Regular physical activity, *n* (%)^f^	708 (53.5)	164 (49.4)	191 (57.7)	167 (50.6)	186 (56.3)	.13
Higher education, *n* (%)^g^	522 (39.4)	128 (38.8)	141 (42.6)	131 (39.7)	122 (37.0)	.82

Data are shown as mean (SD), median (IQR) or *n* (%).

ACR, albumin-creatinine ratio; BMI, body mass index; BP, blood pressure; E%, percentage of total energy intake per day; mGFR, measured glomerular filtration rate.

^a^Hypertension is defined as systolic blood pressure over or equal to 140 mmHg, diastolic blood pressure over or equal to 90 mmHg, or the use of antihypertensive medication.

^b^Defined as absolute GFR (ml/min) above the 90th percentile after adjustment for sex, age, weight, height, and use of renin–angiotensin system inhibitors, corresponding to GFR > 127.1 ml/min.

^c^Defined as either the first occurrence of myocardial infarction, stroke, coronary revascularization procedure without concurrent infarction, diagnosis of stenosis in other arteries, or sudden death without a non-CVD cause.

^d^Fasting plasma glucose 5.6–6.9 mmol/l (100–125 mg/dl), according to the American Diabetes Association definition (35).

^e^BMI ≥ 30 kg/m².

^f^Physical activity is defined as a dichotomous variable, either engaged in 1 or more hours of moderate or vigorous activity per week or less.

^g^Higher education is defined as a dichotomous variable, either college or university education (≥13 years of education) vs high school education or less (<13 years of education).

The mean total protein intake for the cohort was 1.2 ± 0.5 g/kg/day. When divided into quartiles (Q1–Q4), the mean protein intakes were 0.8 ± 0.1 g/kg/day, 1.0 ± 0.1 g/kg/day, 1.3 ± 0.1 g/kg/day, and 1.8 ± 0.5 g/kg/day, respectively. Participants with higher protein intake had lower BMI and systolic blood pressure and fewer cases of hypertension than those with lower protein intake. Baseline characteristics of the complete cases are shown in Supplementary Table S1.

### Associations between protein intake and mean GFR decline rates

The annual change in mGFR per 0.1 g/kg/day increase in total protein intake was −0.01 ml/min/1.73 m² (95% CI: −0.04 to 0.02), in the fully adjusted model (Table 2). There were no significant differences in mGFR decline across the protein intake quartiles. In analyses restricted to participants with less than 12 months between FFQ completion and baseline mGFR measurement (*n* = 281), higher protein intake was not significantly associated with annual mGFR decline in the fully adjusted model (Table 2).

**Table 2:** Linear mixed model regression analysis of the association between total protein intake and annual changes in measured GFR (*n* = 1324) (RENIS).

	Model 1		Model 2		Model 3	
Total protein intake	ml/min per 1.73 m^2^ per year^a^ (95% CI)	*P*-value	ml/min per 1.73 m^2^ per year^a^ (95% CI)	*P*-value	ml/min per 1.73 m^2^ per year^a^ (95% CI)	*P*-value
Per 0.1 g/kg/d increase in protein intake	0.01 (−0.01 to 0.02)	.43	0.00 (−0.03 to 0.03)	.79	−0.01 (−0.04 to 0.02)	.44
Quartile of protein intake^b^						
≤0.8 g/kg/d	Reference		Reference		Reference	
0.9–1.1 g/kg/d	0.01 (−0.18 to 0.19)	.95	−0.06 (−0.25 to 0.13)	.53	−0.13 (−0.32 to 0.07)	.20
1.2–1.3 g/kg/d	0.02 (−0.16 to 0.21)	.80	−0.07 (−0.28 to 0.15)	.54	−0.13 (−0.35 to 0.08)	.23
≥1.4 g/kg/d	0.05 (−0.13 to 0.24)	.59	−0.09 (−0.36 to 0.18)	.51	−0.18 (−0.45 to 0.11)	.23
Participants with less than 12 months between the FFQ and mGFR^c^						
Per 0.1 g/kg/d increase in protein intake	0.02 (−0.03 to 0.08)	.43	0.12 (0.01 to 0.23)	.03	0.07 (−0.05 to 0.19)	.24

CI, confidence interval; g/kg/d, gram/kilogram/day; GFR, glomerular filtration rate.

^a^A negative coefficient indicates a steeper decline.

^b^No linear, quadratic, or cubic trends in mGFR decline were observed across protein-intake quartiles (all *P*-values > .05).

^c^Restricted to participants with less than 12 months between FFQ completion and baseline mGFR measurement (*n* = 281).

Model 1: crude. Model 2: adjusted for age, sex, BMI, and total energy intake. Model 3: Model 2 + adjusted for the proportion of energy from carbohydrates and fats, total sodium intake, systolic blood pressure, use of antihypertensive medication, diabetes mellitus, total cholesterol, glucose levels, urine albumin-creatinine ratio, education, smoking status, and regular physical activity.

No linear, quadratic, or cubic trends in mGFR decline were observed across protein-intake quartiles (all *P*-values > .05). GAMMs indicated no nonlinear association in complete-case analyses. No statistically significant interactions with sex, u-ACR, obesity, hypertension, or prediabetes were found.

### Sensitivity analyses

No significant association was observed using aGFR (Supplementary Table S3). Results were similar after adjusting for baseline GFR (Supplementary Table S4), analysing only participants with diabetes, obesity, or established CVD at baseline (*n* = 117) (Supplementary Table S5), and excluding the 1% lowest and highest protein intake (Supplementary Table S6). Results from complete-case analyses, including only those who completed the FFQ (*n* = 1092), did not differ from those in the imputed dataset (Supplementary Table S7).

### Associations between protein intake and accelerated GFR decline

Increased protein intake was not associated with an accelerated decline in mGFR [OR 0.97 (95% CI: 0.86–1.10) per 0.1 g/kg/day increase] (Table 3). Among participants with two or more GFR measurements (*n* = 1165), the estimates were similar (Supplementary Table S8). Adjusting for baseline mGFR yielded similar outcomes (Supplementary Table S9).

**Table 3:** Associations of protein intake with accelerated measured GFR (mGFR) decline^a^ (RENIS).

	Model 1		Model 2		Model 3	
Total protein intake	OR (95% CI)	*P*-value	OR (95% CI)	*P*-value	OR (95% CI)	*P*-value
Per 0.1 g/kg/d increase in protein intake	0.98 (0.93–1.02)	.32	1.04 (0.93–1.15)	.52	0.97 (0.86–1.10)	.66
Quartile of protein intake						
≤0.8 g/kg/d	Reference		Reference		Reference	
0.9–1.1 g/kg/d	0.83 (0.50–1.39)	.48	1.04 (0.58–1.87)	.91	1.18 (0.61–2.27)	.63
1.2–1.3 g/kg/d	0.71 (0.40–1.25)	.24	1.02 (0.49–2.10)	.96	0.91 (0.43–1.94)	.81
≥1.4 g/kg/d	0.77 (0.46–1.30)	.33	1.34 (0.55–3.30)	.52	1.21 (0.43–3.41)	.71

CI, confidence interval; OR, odds ratio; g/kg/d, gram/kilogram/day.

Model 1: crude. Model 2: adjusted for age, sex, BMI, and total energy intake. Model 3: Model 2 + adjusted for the proportion of energy from carbohydrates and fats, total sodium intake, systolic blood pressure, use of antihypertensive medication, diabetes mellitus, total cholesterol, glucose levels, urine albumin-creatinine ratio, education, smoking status, and regular physical activity.

^a^Defined as the 10% with the steepest mGFR decline rate (< −2.08 ml/min/1.73 m^2^ per year). Multiple imputation (*n* = 1324).

### Associations between protein intake and incident mGFR <60 ml/min/1.73 m²

Among 1064 participants with baseline mGFR ≥60 ml/min/1.73 m², 118 (11.1%) had incident mGFR <60 ml/min/1.73 m² during follow-up. There was no association between higher baseline protein intake and incident mGFR <60 ml/min/1.73 m² [HR 1.09 (95% CI, 0.96–1.21) per 0.1 g/kg/day increase] (Table 4). Model-based cumulative incidence curves by protein-intake quartile are shown in Supplementary Fig. S2. There was no association between baseline protein intake and incident mGFR <60 ml/min/1.73 m² when adjusting for baseline mGFR (Supplementary Table S10).

**Table 4:** Cox interval–censored regression analyses for incident mGFR <60 ml/min/1.73 m² (RENIS).^a^

		Model 1		Model 2		Model 3	
Total protein intake	Events	HR (95% CI)	*P*-value	HR (95% CI)	*P*-value	HR (95% CI)	*P*-value
Per 0.1 g/kg/d increase in protein intake	118	0.94 (0.90 to 0.98)	.02	0.95 (0.87 to 1.03)	.23	1.09 (0.96 to 1.21)	.21
Quartile of protein intake							
≤0.8 g/kg/d	38	Reference		Reference		Reference	
0.9–1.1 g/kg/d	33	0.88 (0.55 to 1.40)	.59	1.22 (0.74 to 2.01)	.43	1.24 (0.78 to 1.99)	.36
1.2–1.3 g/kg/d	20	0.50 (0.29 to 0.86)	.01	0.78 (0.43 to 1.42)	.42	0.79 (0.45 to 1.39)	.42
≥1.4 g/kg/d	27	0.69 (0.42 to 1.12)	.14	1.38 (0.67 to 2.86)	.38	1.37 (0.86 to 2.16)	.18

CI, confidence interval; HR, hazard ratio; g/kg/d, gram/kilogram/day.

Model 1: crude. Model 2: adjusted for age, sex, BMI, and total energy intake. Model 3: Model 2 + adjusted for the proportion of energy from carbohydrates and fats, total sodium intake, systolic blood pressure, use of antihypertensive medication, diabetes mellitus, total cholesterol, glucose levels, urine albumin-creatinine ratio, education, smoking status, and regular physical activity.

^a^Complete case-analysis (*n* = 1064).

## DISCUSSION

In this population-based cohort with repeated mGFR measurements, higher estimated protein intake was not associated with GFR decline among middle-aged and older adults. The narrow confidence intervals around the mGFR slope estimates indicate that increasing protein intake is unlikely to be an effective strategy for preventing CKD.

Previous studies have observed that in individuals without CKD, high protein intake is associated with a faster decline in GFR [19, 20]. Notably, those studies relied on creatinine-based eGFR, which is influenced by non-GFR determinants such as muscle mass and dietary protein intake [25, 26]. Differences from previous eGFR-based studies may partly reflect non-GFR determinants of creatinine. Because we used measured GFR, our findings are less likely to be influenced by these factors. Moreover, mGFR is more sensitive than eGFR in detecting subtle changes, especially in those with near-normal GFR [43]. Our findings are consistent with other observational studies, including the Nurses’ Health Study [24] and the Tehran Lipid and Glucose Study [10], which reported no association between high total protein intake and eGFR decline in individuals with normal kidney function. Nonetheless, susceptibility to CKD associated with higher protein intake may differ by genetics or ethnicity. Particularly, studies in Asian populations have reported faster GFR decline [19, 20].

Although nephron number declines with age even in otherwise healthy kidneys, ageing alone does not necessarily increase single-nephron GFR [5, 6]. However, a lower number of nephrons might reduce renal reserve and potentially increase the risk of hyperfiltration under additional metabolic stressors [6, 16, 44]. Individuals with diabetes, prediabetes, and obesity are at increased risk of hyperfiltration [5, 45]. In our cohort, few participants had diabetes; however, about 20% had obesity, and around 50% had prediabetes at baseline. We observed no interactions between protein intake and obesity, hypertension, prediabetes, or albuminuria for GFR decline risk. These findings were consistent even when analysing only participants with diabetes, obesity, or CVD. Together, these results argue against a clinically relevant effect of habitual protein intake on the decline in kidney function in the absence of CKD, even among individuals susceptible to hyperfiltration.

Despite the growing incidence of CKD [1], evidence-based lifestyle recommendations for the prevention of CKD remain limited [4]. In this population of middle-aged and older adults with largely preserved kidney function, our findings suggest that habitual protein intake within commonly consumed ranges is unlikely to be a major target for preventing reduced mGFR. This is particularly relevant because adequate protein intake is essential for maintaining muscle mass, preventing sarcopenia and frailty, and preserving physical function with ageing [46, 47]. Accordingly, current dietary guidelines, including the new Dietary Guidelines for Americans [15] and the European Society for Clinical Nutrition and Metabolism (ESPEN) guidelines [14], emphasize the need for adequate protein intake, especially for older adults. Other emerging evidence supports these recommendations, as higher protein intake has been associated with lower risk of frailty and mortality in population-based studies [33, 48].

As we examined habitual dietary protein intake but did not assess protein supplementation, it remains unclear whether very high protein intake, as observed among strength-training athletes, may increase CKD risk [13]. Additionally, because our population largely had preserved kidney function at baseline, our findings cannot be extrapolated to individuals with CKD, in whom protein restriction may remain clinically indicated [11].

The main strength of our study is the use of repeated iohexol clearance measurements over ten years, enabling precise evaluation of kidney function independent of non-GFR determinants. Our study has several limitations. First, protein intake was assessed using an FFQ, which may introduce misclassification. The absence of 24-h urinary urea nitrogen measurements limited our ability to objectively validate total protein intake. Nonetheless, this questionnaire has been validated among Norwegian adults against independent reference measures, supporting its use to estimate habitual dietary intake, including total protein intake and the proportion of energy derived from protein [32–34]. Second, the FFQ and iohexol measurements were taken at different times. However, participants were asked to report their dietary habits over the past year, which are unlikely to change substantially, as confirmed by previous studies using FFQ [49]. Third, we lacked data on specific protein sources, which could be important, as plant- and animal-based proteins may affect kidney health differently [9, 10]. We also lacked information on birth weight, which may reflect nephron endowment. Fourth, we adjusted for baseline covariates, but some covariates may have changed during follow-up, introducing regression dilution bias. Nevertheless, because protein intake was assessed at baseline, adjustment for time-updated covariates could lead to overadjustment if these variables were influenced by baseline health status, dietary intake, or kidney function. Finally, our cohort consisted of relatively healthy, middle-aged Norwegian adults aged 55–70 years, which may limit the generalizability of our findings to populations with different dietary patterns or higher CKD prevalence. Further studies in more diverse populations, including older age groups and populations at higher risk of CKD, are needed to confirm the generalizability of these findings.

## CONCLUSION

Our findings from a population-based cohort with repeated iohexol-based GFR measurements over 10 years suggest that higher self-reported protein intake is not associated with GFR decline. These findings indicate that protein restriction may not be a lifestyle strategy for preventing CKD.

## Supplementary Material

sfag235_Supplemental_File

## Data Availability

The data underlying this article cannot be shared publicly due to ethical considerations and participant privacy, as data sharing was not included in the research permissions. The data for this study are stored within secure servers approved by the Regional Ethics Committee. The data can be shared on request as part of a research collaboration. The data can only be accessed directly by members of the research team who have been given access by the data protection officer.
