# Supplementary material for: Higher habitual protein intake is not linked to decline of iohexol-measured GFR in adults ≥40 years
Source: Clin Kidney J. 2026 Jul 11;19(8):sfag235. doi: 10.1093/ckj/sfag235 (PMC13430269; doi:10.1093/ckj/sfag235)
Supplement: sfag235_Supplemental_File [file sfag235_supplemental_file.docx]

# Protein Intake and Long-Term Measured GFR Decline: The Renal Iohexol Clearance Survey - Supplementary

## Supplementary methods

**Iohexol Clearance measurements**

GFR was measured using single-sample plasma iohexol clearance. Serum iohexol concentrations were measured by high-performance liquid chromatography (HPLC) in RENIS-FU and by liquid chromatography-mass spectrometry (LC-MS/MS) in RENIS-3 (Waters Xevo TQ-Class+) and RENIS-4 (Waters Xevo TQ-Absolute). A calibration equation to convert HPLC and LC-MS results was developed and is described previously (1). The mean coefficient of variation for intraindividual mGFR variation was 4.2% (2). Single-sample iohexol clearance has been validated against gold-standard methods (3)

**Iohexol clearance measurements for participants with expected mGFR < 30** **mL/min/1.73 m^2^**

Two studies have suggested differences between single- and multi-sample protocols in patients with low GFR. Tondel et al. found that multi-sample testing was better than single-sample testing for children with mGFR < 30 mL/min/1.73 m^2^ (4). Delanaye et al studied 5106 GFR measurements in adults and found high concordance between the methods, but somewhat lower concordance for GFR< 30 mL/min/1.73 m^2^  (5). As a quality control measure, we measured GFR using both methods for participants with expected mGFR below this threshold in RENIS-4.

The expected mGFR for the participants in RENIS-4 was estimated based on their mGFR measured in the previous RENIS wave, eGFR from clinical visits, and expected GFR decline. We used the European Kidney Function Consortium (EKFC) consensus multi-sample protocol for expected mGFR < 30 mL/min/1.73 m^2^ (6). The participants had both a single sample and samples scheduled at 4, 6, 8, and 10 hours after injection. The single sample was performed as described previous (7), except that we used the single sample optimal time point as one of the multi-sample samples when the difference from the single sample was less than 30 minutes.

Thirteen participants had iohexol clearance measured with both methods. One person was unable to attend the 10-hour sample of the multi-sample protocol and had the last sample taken at 7 hours after injection.

The median (interquartile range) coefficient of determination (R^2^) when log(iohexol-concentration) was regressed on time for each participant was 0.999 (IQR 0.998–0.999). Only one participant had an R^2^ < 0.975, the lower acceptable quality threshold suggested by the EKFC (R^2^ = 0.960).

Supplementary Figure S1 shows a comparison of the results of the two methods. The median (IQR) difference between the single- and multi-sample mGFR (n=13) was 0.16 mL/min/1.73 m^2^ (-0.36 to 0.33). Two participants had differences more than 3 mL/min/1.73 m^2^. They both had mGFR < 20 mL/min/1.73 m^2^ and optimal time points for a single nighttime sample. For logistical reasons, these sampling times had to be delayed until the following morning, which may be the reason why the single sample mGFRs were slightly lower than the multi-sample mGFRs.

**Estimation of individual mGFR slopes**

The mGFR slope for each individual was estimated on the basis of a generalized additive mixed model (GAMM) with mGFR as the dependent variable and time since baseline, sex, BMI, systolic BP, smoking status, fasting glucose, height, body weight and dichotomous variables for individual antihypertensive drugs as the independent variables. Time-dependent variables recorded at each wave of the survey were used. The main effects for time were modeled as separate smooths for each sex. The interactions between the other independent variables and the time variable were included in the model, as were also an interaction between sex and baseline age. An unstructured covariance matrix and random effects for the intercept and slope were included.

mGFR at baseline in RENIS-FU and at the mean follow-up in RENIS-4 were estimated for each participant from this GAMM. For those not participating in RENIS-4 (n=327), the values of the independent variables were carried forward from their last examination. The fixed effect slope for each individual was estimated as the difference between the predicted mGFR at RENIS-4 and RENIS-FU divided by the mean follow-up time. The individual best linear unbiased predictions (BLUPs) of the random slope were added to obtain the total slope estimate for each participant.

References supplementary

1. Eriksen BO, Stefansson VTN, Jenssen TG, et al.; High Ambulatory Arterial Stiffness Index Is an Independent Risk Factor for Rapid Age-Related Glomerular Filtration Rate Decline in the General Middle-Aged Population. Hypertension 2017; 69(4):651–659.

2. Eriksen BO, Stefansson VTN, Jenssen TG, et al.; Elevated blood pressure is not associated with accelerated glomerular filtration rate decline in the general non-diabetic middle-aged population. Kidney Int 2016; 90(2):404–410.

3. Delanaye P, Ebert N, Melsom T, et al.; Iohexol plasma clearance for measuring glomerular filtration rate in clinical practice and research: a review. Part 1: How to measure glomerular filtration rate with iohexol? Clin Kidney J 2016; 9(5):682–99.

4. Tondel C, Salvador CL, Hufthammer KO, et al.; Iohexol plasma clearance in children: validation of multiple formulas and single-point sampling times. Pediatr Nephrol 2018; 33(4):683–696.

5. Delanaye P, Flamant M, Dubourg L, et al.; Single- versus multiple-sample method to measure glomerular filtration rate. Nephrol Dial Transplant 2018; 33(10):1778–1785.

6. Ebert N, Schaeffner E, Seegmiller JC, et al.; Iohexol plasma clearance measurement protocol standardization for adults: a consensus paper of the European Kidney Function Consortium. Kidney Int 2024; 106(4):583–596.

7. Eriksen BO, Mathisen UD, Melsom T, et al.; Cystatin C is not a better estimator of GFR than plasma creatinine in the general population. Kidney Int 2010; 78(12):1305–11.

8. ElSayed NA, Aleppo G, Aroda VR, et al.; 2. Classification and diagnosis of diabetes: standards of care in diabetes—2023. Diabetes care 2023; 46(Supplement_1):S19–S40.

## Supplementary Figures

**Figure S1**. Results of single- and multi-sample iohexol clearance measurements in 13 participants in RENIS-4. The red line represents identity.

**Figure S2.** Model-based cumulative incidence of incident measured GFR <60 mL/min/1.73 m² by protein-intake quartile. Curves were estimated from the interval-censored Cox regression model because the exact timing of crossing the measured GFR threshold was unknown and occurred between study visits. Protein intake was categorized as quartiles of g/kg/day. GFR, glomerular filtration rate.


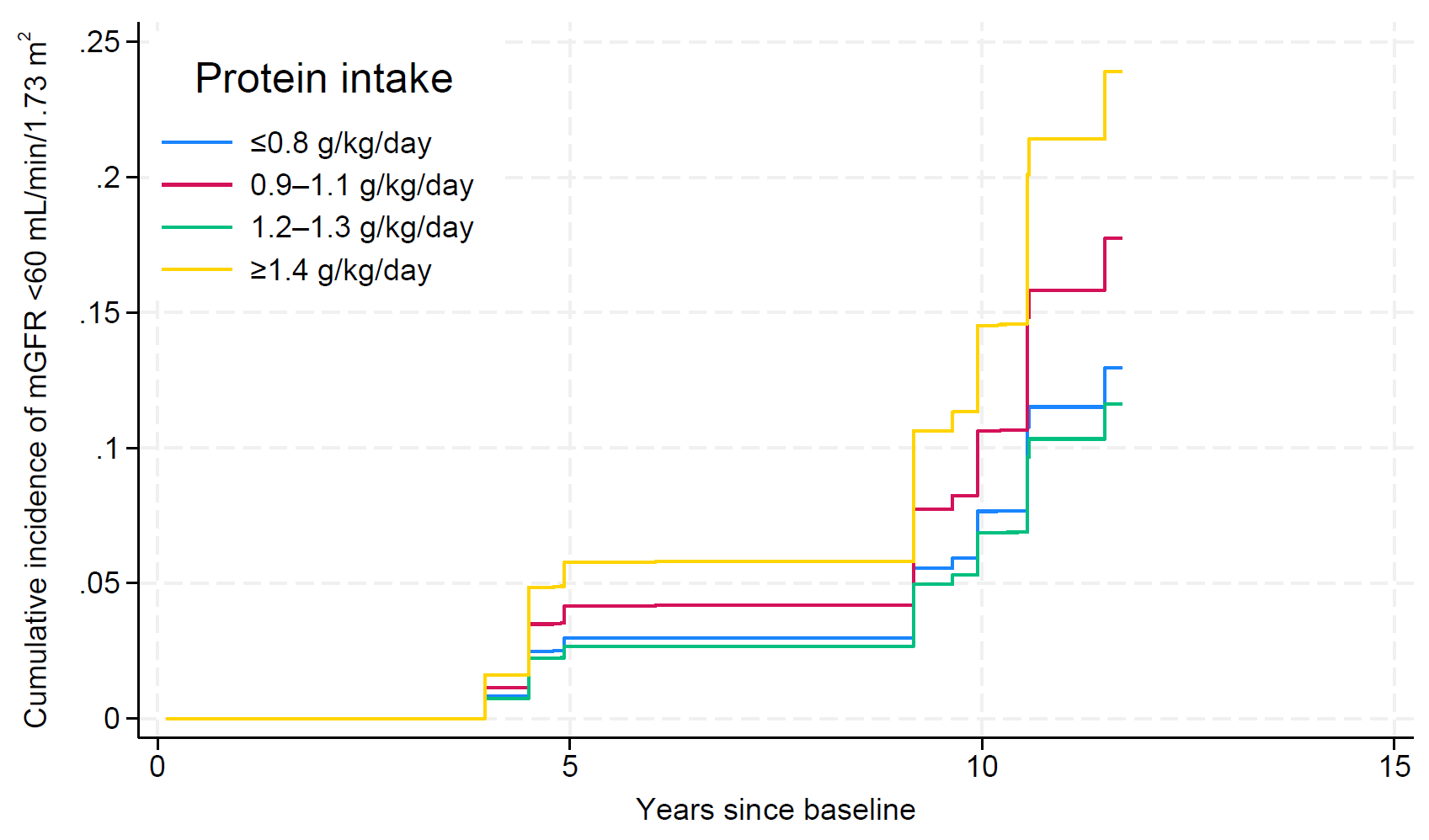


## Supplementary Tables

**Supplementary Table S1.** Imputed variables and the corresponding number of missing baseline observations from the total study population of 1324 in RENIS-FU. The Renal Iohexol Clearance Survey (RENIS).

| Imputed Variables | Missing, n (%) |
| --- | --- |
| Protein g/day | 233 (17.6) |
| Energy intake KJ/day | 233 (17.6) |
| Education | 156 (11.8) |

**Supplementary Table S2.** Baseline Characteristics by Quartiles of Total Protein Intake. Complete case analyses (n = 1091). The Renal Iohexol Clearance Survey (RENIS)

|  |  | Quartile of Total Protein Intake, g/kg/d | | | |  |
| --- | --- | --- | --- | --- | --- | --- |
| Variables | Total  (N = 1,091) | Q1 (<0.9)  (N = 273) | Q2 (0.9 - 1.1)  (N = 273) | Q3 (1.2 - 1.3)  (N = 273) | Q4 (≥1.4)  (N = 272) | P-values |
| Age, Y Mean (SD) | 63.7 (4.0) | 63.6 (4.0) | 63.5 (4.0) | 63.3 (3.9) | 64.3 (4.0) | 0.03 |
| Male, n (%) | 525 (48) | 127 (47) | 142 (52) | 128 (47) | 128 (47) | 0.53 |
| BMI, Mean (SD) | 27.1 (4.0) | 28.9 (4.3) | 27.6 (3.7) | 26.5 (3.5) | 25.3 (3.6) | <0.01 |
| Protein, g/kg, Mean (SD) | 1.2 (0.4) | 0.8 (0.2) | 1.0 (0.1) | 1.3 (0.1) | 1.8 (0.4) | <0.01 |
| Energy, KJ/day, Mean (SD) | 9096.6 (3109.3) | 6447.5 (1720.3) | 8350.2 (1744.1) | 9411.7 (1761.8) | 12188.2 (3524.4) | <0.01 |
| Protein, E%, Mean (SD) | 17.8 (2.7) | 17.0 (3.0) | 17.6 (2.5) | 18.0 (2.3) | 18.5 (2.7) | <0.01 |
| Fat, E%, Mean (SD) | 33.7 (5.9) | 32.4 (7.1) | 33.2 (5.3) | 34.0 (5.4) | 35.1 (5.2) | <0.01 |
| Carbohydrates, E%, Mean (SD) | 42.0 (6.4) | 42.0 (7.8) | 42.5 (5.9) | 42.3 (6.0) | 41.3 (5.8) | 0.11 |
| mGFR, ml/Min/1.73m2, Mean (SD) | 88.9 (14.3) | 85.7 (14.6) | 88.7 (13.6) | 88.9 (14.1) | 92.2 (14.4) | <0.01 |
| Creatinine, µmol/L, Mean (SD) | 70.4 (12.8) | 71.3 (13.0) | 71.4 (13.1) | 70.2 (12.8) | 68.6 (12.0) | 0.04 |
| Urine ACR, Median (IQR) | 0.3 (0.1-0.6) | 0.3(0.1-0.5) | 0.3(0.1-0.6) | 0.3(0.1-0.6) | 0.4 (0.2-0.6) | 0.02 |
| Systolic Blood Pressure, Mean (SD) | 130.7 (16.9) | 133.2 (16.4) | 131.2 (15.5) | 129.0 (17.4) | 129.4 (18.0) | 0.01 |
| Diastolic Blood Pressure, Mean (SD) | 81.9 (9.3) | 83.3 (9.2) | 82.9 (8.6) | 81.1 (9.7) | 80.5 (9.5) | <0.01 |
| Hypertension, n (%) | 569 (52) | 165 (60) | 150 (55) | 131 (48) | 123 (45) | <0.01 |
| mGFR < 60 mL/min/1.73 m^2^, n (%) | 28 (2.6) | 12 (4.4) | 8 (2.9) | 6 (2.2) | 2 (0.7) | 0.06 |
| Cardiovascular disease, n (%)‡ | 44 (4) | 14 (5) | 10 (4) | 13 (5) | 7 (3) | 0.42 |
| Total Cholesterol, Mean (SD) | 5.5 (1.0) | 5.5 (1.0) | 5.5 (0.9) | 5.5 (1.0) | 5.5 (1.0) | 0.84 |
| Diabetes, n (%) | 22 (2) | 7 (3) | 8 (3) | 3 (1.1) | 4 (1.4) | 0.37 |
| Prediabetes, n (%)‡ | 582 (53.5) | 139 (53.5) | 133 (49.5) | 123 (46.0) | 112 (42.3) | <0.01 |
| Obesity, n (%)** | 243 (22.3) | 99 (36.3) | 69 (25.3) | 47 (17.2) | 28 (10.3) | <0.01 |
| Active Smokers, n (%) | 113 (10.4) | 26 (9.6) | 23 (8.5) | 26 (9.6) | 38 (14.1) | 0.16 |
| Physical activity, n (%)\|\| | 604 (56.1) | 134 (50.4) | 155 (57.4) | 159 (58.7) | 156 (58.0) | 0.18 |
| Higher education, n (%)§ | 427 (39.6) | 100 (37.0) | 117 (43.2) | 111 (41.4) | 99 (36.7) | 0.32 |

Data are shown as mean (SD), median (IQR) or n (%).

ACR, albumin-creatinine ratio; BMI, body mass index; BP, blood pressure; E%, percent energy intake per day; mGFR, measured glomerular filtration rate.

*Hypertension is defined as systolic blood pressure over or equal to 140 mmHg, diastolic blood pressure over or equal to 90 mmHg, or the use of antihypertensive medication.

†Defined as either the first occurrence of myocardial infarction, stroke, coronary revascularization procedure without concurrent infarction, diagnosis of stenosis in other arteries, or sudden death without a non-CVD cause

‡Fasting plasma glucose 5.6–6.9 mmol/L [100–125 mg/dL], according to the American Diabetes Association definition (8)

**BMI ≥ 30 kg/m²

||Physical Activity is defined as a dichotomous variable, either engaged in 1 or more hours of moderate or vigorous activity per week or less.

§Higher education is defined as a dichotomous variable, either having completed any length of college or university degree or having completed a high school diploma or less.

**Supplementary Table S3.** Linear Mixed Model Regression Analysis of the Association Between Total Protein Intake and Annual Changes in Absolute mGFR (aGFR) using multiple imputation (n = 1324). The Renal Iohexol Clearance Survey (RENIS)

| Total protein intake | Model 1 | | Model 2 | | Model 3 | |
| --- | --- | --- | --- | --- | --- | --- |
| Annual aGFR decline rates | ml/min per yr ^a^ (95% CI) | P-value | ml/min per yr ^a^ (95% CI) | P-value | ml/min per yr ^a^ (95% CI) | P-value |
| Per 0.1 g/kg/d increase in protein intake | 0.02 (-0.001 to 0.03) | 0.07 | 0.02 (-0.01 to 0.06) | 0.20 | 0.01 (-0.02 to 0.05) | 0.38 |
| Quartile of protein intake |  |  |  |  |  |  |
| ≤ 0.8 g/kg/d | Reference |  | Reference |  | Reference |  |
| 0.9 - 1.1 g/kg/d | 0.04 (-0.17 to 0.26) | 0.69 | 0.02 (-0.23 to 0.20) | 0.86 | -0.08 (-0.30 to 0.14) | 0.48 |
| 1.2 - 1.3 g/kg/d | 0.10 (-0.11 to 0.32) | 0.35 | 0.03 (-0.21 to 0.26) | 0.84 | -0.04 (-0.39 to 0.20) | 0.74 |
| ≥ 1.4 g/kg/d | 0.20 (-0.02 to 0.41) | 0.07 | 0.10 (-0.20 to 0.41) | 0.51 | 0.03 (-0.29 to 0.35) | 0.85 |

CI, confidence interval; g/kg/d, gram/kilogram/day; GFR, glomerular filtration rate

^a^A negative coefficient indicates a steeper decline.

Model 1: crude, Model 2: adjusted for age, sex, BMI, and total energy intake, Model 3: Model 2 + adjusted for the proportion of energy from carbohydrates and fats, total sodium intake, systolic blood pressure, use of antihypertensive medication, diabetes mellitus, total cholesterol, glucose levels, urine albumin-creatinine ratio, education, smoking status, and regular physical activity.

**Supplementary Table S4.** Linear Mixed Model Regression Analysis of the Association Between Total Protein Intake and Annual Changes in mGFR using multiple imputation (n = 1324) adjusting for baseline GFR. The Renal Iohexol Clearance Survey (RENIS)

| Total protein intake | Model 1 | | Model 2 | | Model 3 | |
| --- | --- | --- | --- | --- | --- | --- |
| Annual mGFR decline rates | ml/min per yr ^a^ (95% CI) | P-value | ml/min per yr ^a^ (95% CI) | P-value | ml/min per yr ^a^ (95% CI) | P-value |
| Per 0.1 g/kg/d increase in protein intake | 0.01 (-0.01 to 0.04) | 0.24 | 0.02 (-0.01 to 0.05) | 0.22 | -0.00 (-0.04 to 0.03) | 0.86 |
| Quartile of protein intake |  |  |  |  |  |  |
| ≤ 0.8 g/kg/d | Reference |  | Reference |  | Reference |  |
| 0.9 - 1.1 g/kg/d | 0.08 (-0.12 to 0.27) | 0.44 | 0.01 (-0.20 to 0.21) | 0.96 | -0.07 (-0.29 to 0.14) | 0.49 |
| 1.2 - 1.3 g/kg/d | 0.13 (-0.07 to 0.32) | 0.20 | 0.04 (-0.18 to 0.28) | 0.69 | -0.03 (-0.26 to 0.21) | 0.82 |
| ≥ 1.4 g/kg/d | 0.23 (0.03 to 0.43) | 0.02 | 0.12 (-0.16 to 0.41) | 0.40 | -0.02 (-0.32 to 0.29) | 0.92 |

CI, confidence interval; g/kg/d, gram/kilogram/day; GFR, glomerular filtration rate

^a^A negative coefficient indicates a steeper decline.

Model 1: adjusted for baseline mGFR

Model 2: Model 1 + adjusted for age, sex, BMI, and total energy intake,

Model 3: Model 2 + adjusted for the proportion of energy from carbohydrates and fats, total sodium intake, systolic blood pressure, use of antihypertensive medication, diabetes mellitus, total cholesterol, glucose levels, urine albumin-creatinine ratio, education, smoking status, and regular physical activity.

**Supplementary Table S5.** Linear Mixed Model Regression Analysis of the Association Between Total Protein Intake and Annual Changes in Measured GFR (mGFR) with individuals with diabetes, obesity, and established CVD at baseline, multiple imputed (n = 117) The Renal Iohexol Clearance Survey (RENIS)

| Total protein intake | Model 1 | | Model 2 | | Model 3 | |
| --- | --- | --- | --- | --- | --- | --- |
|  | ml/min per 1.73 m^2^ per yr ^a^ (95% CI) | P-value | ml/min per 1.73 m^2^ per yr ^a^ (95% CI) | P-value | ml/min per 1.73 m^2^ per yr ^a^ (95% CI) | P-value |
| Per 0.1 g/kg/d increase in protein intake | 0.01 (-0.01 to 0.02) | 0.49 | -0.00 ( -0.03 to 0.03) | 0.96 | -0.01 (-0.03 to 0.02) | 0.71 |
| Quartile of protein intake |  |  |  |  |  |  |
| ≤ 0.8 g/kg/d | Reference |  | Reference |  | Reference |  |
| 0.9 - 1.1 g/kg/d | 0.01 (-0.18 to 0.20) | 0.91 | -0.03 (-0.22 to 0.17) | 0.77 | -0.08 (-0.28 to 0.12) | 0.43 |
| 1.2 - 1.3 g/kg/d | 0.05 (-0.14 to 0.23) | 0.63 | -0.02 (-0.23 to 0.19) | 0.86 | -0.05 (-0.27 to 0.16) | 0.62 |
| ≥ 1.4 g/kg/d | 0.06 (-0.13 to 0.24) | 0.56 | 0.03 (-0.30 to 0.23) | 0.80 | -0.08 (-0.36 to 0.19) | 0.55 |

CI, confidence interval; CVD, cardiovascular disease, g/kg/d, gram/kilogram/day; GFR, glomerular filtration rate

^a^A negative coefficient indicates a steeper decline.

Model 1: crude

Model 2: adjusted for age, sex, BMI, and total energy intake

Model 3: Model 2 + adjusted for the proportion of energy from carbohydrates and fats, total sodium intake, systolic blood pressure, use of antihypertensive medication, diabetes mellitus, total cholesterol, glucose levels, urine albumin-creatinine ratio, education, smoking status, and regular physical activity.

**Supplementary Table S6.** Linear Mixed Model Regression Analysis of the Association Between Total Protein Intake and Changes in Measured (mGFR) at baseline, excluding those with the 1% lowest and 1% highest protein intake, multiple imputed (n=1309). The Renal Iohexol Clearance Survey (RENIS)

| Total protein intake | Model 1 | | Model 2 | | Model 3 | |
| --- | --- | --- | --- | --- | --- | --- |
|  | ml/min per 1.73 m^2^ per yr ^a^ (95% CI) | P-value | ml/min per 1.73 m^2^ per yr ^a^ (95% CI) | P-value | ml/min per 1.73 m^2^ per yr ^a^ (95% CI) | P-value |
| Per 0.1 g/kg/d increase in protein intake | 0.004 (-0.01 to 0.02) | 0.62 | -0.00 (-0.03 to 0.03) | 0.81 | -0.01 (-0.04 to 0.02) | 0.45 |
| Quartile of protein intake |  |  |  |  |  |  |
| ≤ 0.8 g/kg/d | Reference |  | Reference |  | Reference |  |
| 0.9 - 1.1 g/kg/d | -0.02 (-0.21 to 0.17) | 0.83 | -0.07 (-0.27 to 0.12) | 0.45 | -0.14 (-0.33 to 0.06) | 0.17 |
| 1.2 - 1.3 g/kg/d | -0.02 (-0.19 to 0.19) | 0.98 | -0.07 (-0.19 to 0.14) | 0.49 | -0.14 (-0.36 to 0.06) | 0.21 |
| ≥ 1.4 g/kg/d | 0.03 ( -0.16 to 0.21) | 0.79 | -0.09 (-0.36 to 0.18) | 0.53 | -0.17 (-0.44 to 0.11) | 0.25 |

CI, confidence interval; g/kg/d, gram/kilogram/day; GFR, glomerular filtration rate

^a^A negative coefficient indicates a steeper decline.

Model 1: crude

Model 2: adjusted for age, sex, BMI, and total energy intake

Model 3: Model 2 + adjusted for the proportion of energy from carbohydrates and fats, total sodium intake, systolic blood pressure, use of antihypertensive medication, diabetes mellitus, total cholesterol, glucose levels, urine albumin-creatinine ratio, education, smoking status, and regular physical activity.

**Supplementary Table S7.** Linear Mixed Model Regression Analysis of the Association Between Total Protein Intake and Annual Changes in Measured GFR (mGFR) using complete case analyses (n = 1091). The Renal Iohexol Clearance Survey (RENIS).

| Total protein intake | Model 1 | | Model 2 | | Model 3 | |
| --- | --- | --- | --- | --- | --- | --- |
|  | ml/min per 1.73 m^2^ per yr ^a^ (95% CI) | P-value | ml/min per 1.73 m^2^ per yr ^a^ (95% CI) | P-value | ml/min per 1.73 m^2^ per yr ^a^ (95% CI) | P-value |
| Per 0.1 g/kg/d increase in protein intake | 0.01 (-0.01 to 0.02) | 0.53 | -0.01 ( -0.04 to 0.02) | 0.55 | -0.01 ( -0.04 to 0.03) | 0.70 |
| Quartile of protein intake |  |  |  |  |  |  |
| ≤ 0.8 g/kg/d | Reference |  | Reference |  | Reference |  |
| 0.9 - 1.1 g/kg/d | -0.05 (-0.24 to 0.14) | 0.62 | -0.13 ( -0.33 to 0.07) | 0.19 | -0.15 ( -0.34 to 0.05) | 0.13 |
| 1.2 - 1.3 g/kg/d | 0.01 (-0.18 to 0.21) | 0.90 | -0.13 ( -0.35 to 0.09) | 0.26 | -0.14 (-0.36 to 0.08) | 0.20 |
| ≥ 1.4 g/kg/d | 0.00 (-0.19 to 0.19) | 1.00 | -0.21 ( -0.50 to 0.08) | 0.16 | -0.18 ( -0.46 to 0.10) | 0.22 |

CI, confidence interval; g/kg/d, gram/kilogram/day; GFR, glomerular filtration rate

^a^A negative coefficient indicates a steeper decline.

Model 1: crude

Model 2: adjusted for age, sex, BMI, and total energy intake

Model 3: Model 2 + adjusted for the proportion of energy from carbohydrates and fats, total sodium intake, systolic blood pressure, use of antihypertensive medication, diabetes mellitus, total cholesterol, glucose levels, urine albumin-creatinine ratio, education, smoking status, and regular physical activity.

**Supplementary Table S8.** Associations of protein intake with accelerated GFR decline, defined as the 10% with the steepest GFR decline rate (-2.08 ml/min per 1.73 m2 per year) in only participants with two or more GFR measurements. Multiple imputation (n = 1165). The Renal Iohexol Clearance Survey (RENIS)

| Total protein intake | Model 1 | | Model 2 | | Model 3 | |
| --- | --- | --- | --- | --- | --- | --- |
|  | OR (95% CI) | P-value | OR (95% CI) | P-value | OR (95% CI) | P-value |
| Per 0.1 g/kg/d increase in Protein Intake | 0.95 (0.90–1.00) | 0.07 | 0.99 (0.87–1.12) | 0.85 | 0.94 (0.79–1.12) | 0.48 |
| Quartile of protein intake |  |  |  |  |  |  |
| <0.8 g/kg/d | Reference |  | Reference |  | Reference |  |
| 0.9 - 1.1 g/kg/d | 0.72 (0.41–1.26) | 0.25 | 0.97 (0.51–1.83) | 0.92 | 1.04 (0.51–2.13) | 0.91 |
| 1.2 - 1.3 g/kg/d | 0.59 (0.32–1.07) | 0.08 | 0.86 (0.40–1.88) | 0.71 | 0.73 (0.32–1.70) | 0.47 |
| >1.4 g/kg/d | 0.60 (0.34–1.05) | 0.07 | 1.05 (0.39–2.82) | 0.92 | 1.01 (0.30–3.38) | 0.99 |

CI, confidence interval; OR, odds ratio; g/kg/d, gram/kilogram/day

Model 1: crude

Model 2: adjusted for age, sex, BMI, and total energy intake

Model 3: Model 2 + adjusted for the proportion of energy from carbohydrates and fats, total sodium intake, systolic blood pressure, use of antihypertensive medication, diabetes mellitus, total cholesterol, glucose levels, urine albumin-creatinine ratio, education, smoking status, and regular physical activity.

*Defined as the 10% with the steepest mGFR decline rate (< -2.08 ml/min per 1.73 m^2^ per year)

**Supplementary Table S9.** Associations of protein intake with accelerated GFR decline, defined as the 10% with the steepest GFR decline rate (-2.08 ml/min per 1.73 m^2^ per year) adjusted for baseline mGFR. Multiple imputation (n = 1324). The Renal Iohexol Clearance Survey (RENIS)

| Total protein intake | Model 1 | | Model 2 | | Model 3 | |
| --- | --- | --- | --- | --- | --- | --- |
|  | OR (95% CI) | P-value | OR (95% CI) | P-value | OR (95% CI) | P-value |
| Per 0.1 g/kg/d increase in Protein Intake | 0.95 (0.91-1.00) | 0.07 | 0.99 (0.88-1.11) | 0.83 | 0.94 (0.82-1.08) | 0.37 |

CI, confidence interval; mGFR, measured glomerular filtration rate; OR, odds ratio; g/kg/d, gram/kilogram/day

Model 1: baseline mGFR

Model 2: Model 1 + adjusted for age, sex, BMI, and total energy intake

Model 3: Model 2 + adjusted for the proportion of energy from carbohydrates and fats, total sodium intake, systolic blood pressure, use of antihypertensive medication, diabetes mellitus, total cholesterol, glucose levels, urine albumin-creatinine ratio, education, smoking status, and regular physical activity.

**Supplementary Table S10.** Cox interval–censored regression analyses for new-onset chronic kidney disease with measured glomerular filtration rate < 60 ml/min per 1.73 m² in study participants with GFR ≥ 60 ml/min per 1.73 m² adjusted for baseline mGFR. Complete case-analyses (n = 1064). The Renal Iohexol Clearance Survey (RENIS)

| Total protein intake | Events of CKD | Model 1 | | Model 2 | | Model 3 | |
| --- | --- | --- | --- | --- | --- | --- | --- |
|  |  | HR (95% CI) | P-value | HR (95% Confidence Interval) | P-value | HR (95% Confidence Interval) | P-value |
| Per 0.1 g/kg/d increase in protein intake | 118 | 0.98 (0.93 to 1.02) | 0.30 | 1.01 (0.92 to 1.11) | 0.77 | 1.07 (0.96 to 1.21) | 0.21 |
| Quartile of protein intake | | |  |  |  |  |  |
| ≤ 0.8 g/kg/d | 38 | Reference |  | Reference |  | Reference |  |
| 0.9 - 1.1 g/kg/d | 33 | 1.25 (0.80 to 1.94) | 0.33 | 1.59 (1.04 to 2.42) | 0.03 | 1.61 (0.97 to 2.66) | 0.06 |
| 1.2 - 1.3 g/kg/d | 20 | 0.63 (0.37 to 1.07) | 0.09 | 0.99 (0.83 to 1.19) | 0.42 | 1.02 (0.54 to 1.92) | 0.95 |
| ≥ 1.4 g/kg/d | 27 | 1.09 (0.67 to 1.79) | 0.72 | 2.30 (1.16 to 4.53) | 0.02 | 2.06 (0.94 to 4.54) | 0.07 |

CI, confidence interval; mGFR, measured glomerular filtration rate; HR, hazard ratio; g/kg/d, gram/kilogram/day

Model 1: baseline mGFR

Model 2: Model 1 + adjusted for age, sex, BMI, and total energy intake

Model 3: Model 2 + adjusted for the proportion of energy from carbohydrates and fats, total sodium intake, systolic blood pressure, use of antihypertensive medication, diabetes mellitus, total cholesterol, glucose levels, urine albumin-creatinine ratio, education, smoking status, and regular physical activity.
